# Analysis of IgM, IgA, and IgG isotype antibodies Directed against SARS-CoV-2 spike glycoprotein and ORF8 in the course of COVID-19

**DOI:** 10.1038/s41598-021-88356-8

**Published:** 2021-04-26

**Authors:** Denise Meinberger, Manuel Koch, Annika Roth, Gabriele Hermes, Jannik Stemler, Oliver A. Cornely, Thomas Streichert, Andreas R. Klatt

**Affiliations:** 1 Institute for Clinical Chemistry, Medical Faculty, University of Cologne, 50937 Cologne, Germany; 2 Institute for Dental Research and Oral Musculoskeletal Biology, Medical Faculty, University of Cologne, 50931 Cologne, Germany; 3 Center for Biochemistry II, Medical Faculty, University of Cologne, 50931 Cologne, Germany; 4 Center for Molecular Medicine Cologne, Medical Faculty, University of Cologne, 50931 Cologne, Germany; 5 Department I of Internal Medicine, Excellence Center for Medical Mycology (ECMM), Medical Faculty, University of Cologne, 50937 Cologne, Germany; 6 Chair Translational Research, Cologne Excellence Cluster On Cellular Stress Responses in Aging-Associated Diseases (CECAD), Medical Faculty, University of Cologne, 50931 Cologne, Germany; 7 German Centre for Infection Research (DZIF), Partner Site Bonn-Cologne, Medical Faculty, University of Cologne, 50937 Cologne, Germany

**Keywords:** Viral infection, Antibodies

## Abstract

Immunoassays are a standard diagnostic tool that assesses immunity in severe acute respiratory syndrome coronavirus type 2 (SARS-CoV-2) infection. However, immunoassays do not provide information about contaminating antigens or cross-reactions and might exhibit inaccurately high sensitivity and low specificity. We aimed to gain insight into the serological immune response of SARS-CoV-2 patients by immunoblot analysis. We analyzed serum immunoglobulins IgM, -A, and -G directed against SARS-CoV-2 proteins by immunoblot analysis from 12 infected patients. We determined IgG isotype antibodies by commercially available ELISA and assessed the clinical parameters of inflammation status and kidney and liver injury. Unexpectedly, we found no correlation between the presence of antibodies and the future course of the disease. However, attention should be paid to the parameters CRP, IL-6, and LDH. We found evidence of antibody cross-reactivity, which questions the reliability of results for serum samples that tested negative for anti-SARS-CoV-2 antibodies when assessed by immunoassays. Nevertheless, for the detection of IgG anti-SARS-CoV-2 antibodies, our data suggest that the use of the spike glycoprotein in immunoassays should be sufficient to identify positive patients. Using a combination of the spike glycoprotein and the open reading frame 8 protein could prove to be the best way of detecting anti-SARS-CoV-2 IgM antibodies.

## Introduction

Severe acute respiratory syndrome coronavirus type 2 (SARS-CoV-2) is one of seven known human coronavirus pathogens. Most human coronavirus infections result in the common cold and account for up to 15% of such cases^1^. In contrast, SARS-CoV, MERS-CoV, and SARS-CoV-2 infections can develop into life-threatening lower respiratory syndromes^2–4^. As of October 23, 2020, a total of 42,089,893 infected cases and more than 1,144,274 deaths (mortality ~ 4%) were reported (WorldOmeter https://www.worldometers.info/coronavirus/). Coronavirus disease 2019 (COVID-19) has resulted in a global pandemic.

Coronaviruses are single-stranded RNA viruses. The SARS-CoV-2 genome encodes few proteins despite its large size of approximately 30,000 bases. The structural proteins are spike, nucleocapsid, membrane, and envelope proteins, which are needed to produce structurally complete viral particles^5^. Additionally, 16–17 non-structural proteins (ns1 to ns17) are coded for, such as 3-chymotrypsin-like protease, papain-like protease, helicase, RNA-dependent RNA polymerase^5^, and multiple uncharacterized proteins encoded by open reading frame (ORF) 3a, ORF6, ORF7, ORF8, and ORF10. ORF8 and several proteins from alpha- and beta-coronaviruses define novel families of immunoglobulin (Ig) domains which might function as potential immune modulators that delay or attenuate the host immune response against the viruses^6^.

Accurate and fast diagnosis of SARS-CoV-2 infection is important to identify COVID-19 patients, limit the extent of the pandemic, and initiate therapeutic measures. Polymerase chain reaction (PCR) is the standard diagnostic tool. Immunoassays are essential for assessing the immunity of patients post-infection and analyzing the efficacy of vaccines. Various serological immunoassays have been rapidly developed^7^. The spike glycoprotein of SARS-CoV-2 contains immunodominant epitopes and was used as an antigen in immunoassays to identify antibodies directed against SARS-CoV-2, providing high specificity and sensitivity^8, 9^. The diagnostic performance of commercially available SARS-CoV-2 immunoassays is particularly good, and the diagnostic sensitivity and specificity are generally higher than 80% or even 90%. However, immunoassays do not provide information about cross-reactivity or contaminating antigens. This may lead to lower specificity and false high sensitivity. While the number of studies employing immunoassays is rapidly increasing, no study has examined antibody response using more specific methods, namely, immunoblots.

We used immunoblot analysis to investigate the serum of 12 COVID-19 patients with mild, moderate, or severe disease for the presence of IgM, -A, and -G isotype antibodies directed towards SARS-CoV-2 spike protein (S), receptor-binding domain (RBD) of SARS-CoV-2 spike protein (R), subunit 1 of the spike protein (S1), and ORF8 protein (O). Furthermore, we investigated each patient’s serum using a commercially available anti-S1 IgG isotype ELISA. C-reactive protein (CRP), procalcitonin (PCT), and interleukin-6 (IL-6) were determined to assess the systemic inflammatory reaction of the patients.

## Material and methods

### Study and ethics

This case–control study was performed at and under the guidelines of the University Hospital of Cologne and approved by the ethics committee of the Medical Faculty of the University of Cologne (ISI protocol, ID 08-160). Written informed consent was obtained from all patients and controls.

### Demographic and clinical characteristics

The study included 12 patients who tested positive for SARS-CoV-2 infection by PCR. Four showed mild symptoms not requiring oxygen supply (WHO 3), six displayed moderate symptoms requiring non-invasive oxygen supply (WHO 4), and two developed severe symptoms requiring invasive ventilation (WHO 5–7)^10^. The latter patients each died of an underlying chronic disease. The age of the patients with a mild or moderate course of COVID-19 ranged from 31 to 89 years with a median age of 59 years; 50% were female. The age of the patients with a severe course was between 65 and 88 years, with a median age of 77 years; 50% were female in this group. The predisposing conditions were hypertension, diabetes, surgery, malignancies, chronic lung disease, and chronic renal diseases. As controls, the serum of three donors without SARS-CoV-2 infection was used. The age of these all-male donors ranged from 30 to 50 years with a median age of 38 years.

### Blood samples

Samples were collected between March 27 and May 11, 2020, in 4.7-ml serum monovettes (REF 03.1524, Sarstedt, Germany), centrifuged at 2772 g for 10 min, and stored at – 80 °C.

### Cloning and recombinant expression of SARS-CoV-2 proteins

Spike ectodomain (138.8 kDa) (MN908947; AA: 1-1207), including a C-terminal T4 foldon (AA: GSGYIPEAPRDGQAYVRKDGEWVLLSTFLRSL), RBD (26 kDa) (MN908947; AA: 331-524), and subunit 1 (62.7 kDa) (MN908947; AA: 14-529) regions of the spike DNA, as well as ORF8 (16.3 kDa) (MN908947; AA: 16-121), were amplified from synthetic gene plasmids (furin site mutated, K986P and V987P; GeneArt Gene Synthesis by Thermo Fisher Scientific) using specific PCR primers (Supplementary Table S1). PCR products were digested with the appropriate restriction enzymes (Supplementary Table S1) and cloned into modified Sleeping Beauty transposon expression vectors^11^ digested with NheI and BamHI. To facilitate the protein purification a BM40 signal peptide sequence followed by a twin strep tag was introduced at the N-terminus (AA:MRAWIFFLLCLAGRALAAPLESWSHPQFEKGGGSGGGSGGGWSHPQFEKSGLVPRG). In the case of the spike ectodomain, the signal peptide from the virus was used and the twin strep tag was introduced at the C-terminus. Gene-optimized DNA sequences from the different constructs can be obtained upon request (Manuel.Koch@uni-koeln.de). The four expression constructs were transfected into HEK293 EBNA cells using FuGENE® HD (Promega GmbH, USA) in DMEM/F12 (Merck, Germany) supplemented with 6% fetal bovine serum (Biochrom AG, Germany) in 6-well plates. After 24 h the cells were selected with puromycin (3 µg/ml; Sigma, USA) for four days. Each cell line was expanded in one 10-cm dish and afterward transferred into a Nunc™ TripleFlask™ (VWR, Germany) for protein production. After reaching confluency (5–7 days) cells were induced with doxycycline hyclate (0.5 µg/ml, Sigma, USA) in DMEM/F12 (Merck, Germany) supplemented with 2% fetal bovine serum (Biochrom AG, Germany). Cell supernatants were harvested every third day and replaced with fresh induction medium. Around 500 ml of supernatants were filtered and the recombinant proteins were applied onto a Strep-Tactin®XT (IBA Lifescience, Germany) column. After an extensive TBS and high salt wash step (1 M NaCl/TBS), proteins were eluted with biotin elution buffer (IBA Lifescience, Germany), dialyzed against Tris- or phosphate-buffered saline (TBS/PBS), and stored at 4 °C.

### SDS-PAGE and immunoblot

**Figure 1:**
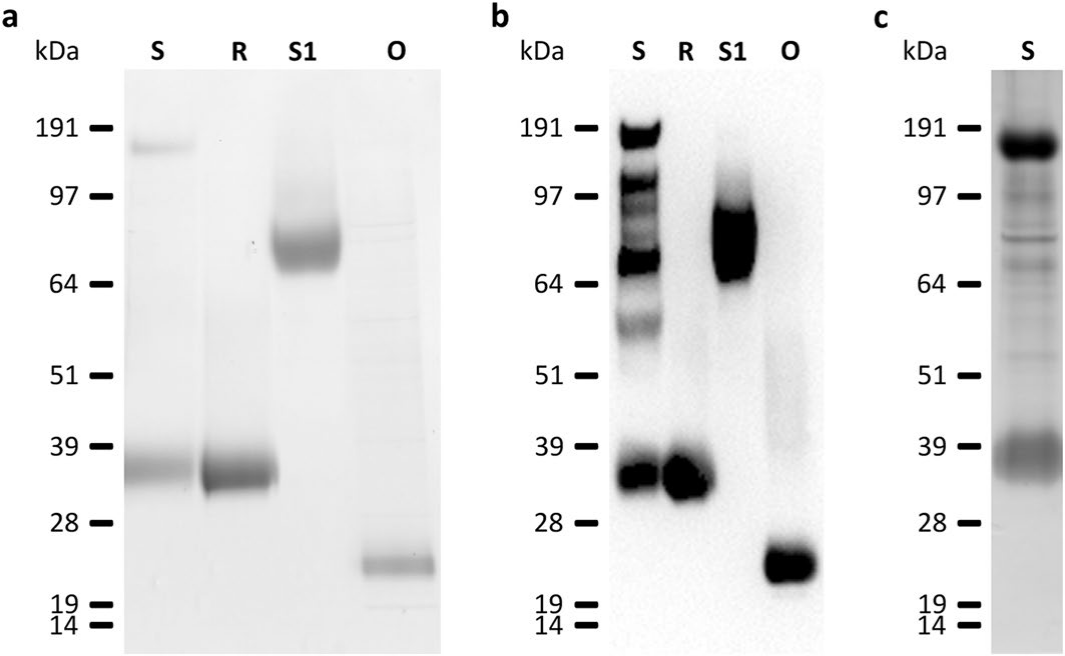
Recombinant SARS-CoV-2 proteins. (**a**) 4–12% Bis–Tris gel loaded with 4 µg of SARS-CoV-2 spike protein (S), the RBD of spike protein (R), subunit 1 of the spike protein (S1), and ORF8 protein (O) stained with Coomassie brilliant blue R 250. (**b**) Immunoblot with 2 µg of Strep-tagged SARS-CoV-2 proteins loaded in the same manner as the Coomassie gel in (**a**), and detected with a Strep-Tactin-HRP conjugate. (**c**) Silver staining with 2 µg of SARS-CoV-2 S protein loaded in the same manner as the Coomassie gel in (**a**). All cropped lanes of different immunoblots or gels are divided by white space. Immunoblots and gels were only cut in width and lanes are shown in their full length.

Recombinant SARS-CoV-2 proteins were separated on 4–12% Bis–Tris polyacrylamide gels (Thermo Fisher Scientific, USA) and stained with Coomassie brilliant blue R-250 (Merck, Germany) (Fig. 1a) or using the SilverQuest silver staining kit (Invitrogen Thermo Fisher Scientific, USA) (Fig. 1c). The proteins were transferred to a polyvinylidene fluoride (PVDF, 0.45 μm) membrane (Thermo Fisher Scientific, USA). For the Strep-Tactin®-HRP conjugate (IBA Lifescience, Germany), membranes were blocked in PBS containing 0.05% Tween 20 and 3% BSA (PBS-TB) and incubated with Strep-Tactin®-HRP conjugate diluted 1:100,000 in PBS containing 0.05% Tween 20 (PBS-T). Otherwise, membranes were blocked in TBS containing 0.01% Tween 20 (Merck, Germany), 5% milk powder (Roth, Germany), and 1% bovine serum albumin (Sigma, USA) (TBS-TMB) and incubated with the patients´ serum diluted 1:200 in TBS-TMB. Finally, the membranes were incubated with cross-adsorbed HRP-conjugated polyclonal goat anti-human IgG (1:50,000), IgM (1:50,000), and IgA (1:20,000) (A18841, A18829 Novex Thermo Fisher Scientific, USA; ab98558 Abcam, England) in TBS-TMB. Membranes were treated with Amersham™ ECL™ Prime Western Blotting Detection Reagent (Thermo Fisher Scientific, USA), and signals were visualized with the ChemiDoc XRS + System (BioRad, Germany). Signals were analyzed using Image Lab software version 4.0 from BioRad. Patients were considered positive for anti-SARS-CoV-2 antibodies if their signals exceeded the strongest signal of the controls. Prestained SeeBlue™ Plus 2 protein standard (Thermo Fisher Scientific, USA) was used for immunoblotting and Coomassie staining.

### Enzyme-linked immunosorbent binding assay (ELISA)

ELISA was performed with an IDK® anti-SARS-CoV-2 IgG ELISA kit from Immundiagnostik AG (Germany) strictly following the provided protocol. Pre-coated and blocked 96-well plates were incubated with 100 µl of patient serum diluted 1:101 for 1 h under shaking at 900 rpm. After five washing steps with the provided wash buffer, bound human IgG was detected with the provided conjugate. After another five washes, a color reaction was obtained using the provided substrate and stopped with stop solution. Absorbance was detected at 450 nm using a TECAN microplate reader (Tecan Life Science, Switzerland). Samples were measured in duplicate. Patients were considered positive for anti-SARS-CoV-2 antibodies if their signals were stronger than those of the provided cut-off control.

## Results

### Clinical characteristics and laboratory data

The patients’ conditions regarding their respiratory symptoms were clinically classified into mild (P1–P4), moderate (P5–P10), and severe courses of the disease (P11 and P12) (Table 1). Additionally, we determined the serum levels of CRP and IL-6, as standard inflammation markers for assessing the systemic inflammatory response of the patients, and of PCT, as a parameter that helps to discriminate between bacterial and non-bacterial infections. We used serum samples taken within the first 72 h of hospital admission (day 1–3) and approximately one week later (day 6–10) (Table 1). Except for P3, all patients with a mild or moderate course of COVID-19 (P1–10) had very high CRP (> 100 mg/l) levels on admission, indicative of generalized high-grade inflammation; however, the CRP levels of these patients decreased after one week. In contrast, P11 and P12, who suffered from severe courses of COVID-19, had CRP levels below 100 mg/l on admission, but after one week the CRP level of P12 increased to over 100 mg/l, while the CRP level of P11 decreased. In contrast to CRP, PCT serum levels did not increase in most patients. Only P9 and P11 showed PCT serum levels of approximately 2 µg/ml, suggesting an additional systemic bacterial infection. Upon admission to the hospital, the IL-6 levels of nine patients were below 150 ng/l, indicative of local inflammation, such as happens during pneumonia. Only P9 and P11 had IL-6 levels higher than 150 ng/l, suggesting a systemic inflammatory response. However, one week later the IL-6 levels of all patients decreased, except for P12, who showed an increase above 150 ng/l. Interestingly, 11 out of 12 patients showed elevated CRP serum levels, but only one out of these 11 patients (P8) showed leucocyte counts above the reference range. Conversely, P3 had no elevated CRP level but an elevated leukocyte count. Nine of the 12 patients showed lymphocyte counts below the reference range. We also determined three further parameters: serum creatinine, to estimate the glomerular filtration rate (which correlates to renal function); the transaminases aspartate aminotransferase (ASAT) and alanin aminotransferase (ALAT), which are released by hepatocytes upon liver damage; and lactate dehydrogenase (LDH), to assess hemolysis and cell damage (Table 1). P9 and P11 had elevated creatinine levels associated with preexisting renal failure. Some patients showed elevated transaminases, but there was no evidence of acute liver failure. The calculated averages for LDH rose in line with the severity of the disease, with 256 U/l at day 1 for the mild cohort, 573 U/l for the moderate cohort, and 473 U/l for the severe cohort. While the average levels of LDH declined after seven days in the mild and moderate cohorts to 229 U/l and 397 U/l, respectively, the LDH level of the severe cohort increased to 622 U/l.

**Table 1:** Clinical parameters of the patients.

Patient Nr	Sex Age	Days since admission	CRP [mg/l]	PCT [µg/l]	IL-6 [ng/l]	Leucocytes [1E9/l]	Lymphocytes [1E9/l]	Creatinine [mg/dl]	ASAT [U/l]	ALAT [U/l]	LDH [U/l]	LDH average
**Mild CoD WHO 3**												
1	W 31	1	282.3	0.2	89	1.7	0.36	0.46	18	12	188	Day 1 256
		7	104.4	0.1	21	3.6	0.37	0.45	33	29	182	
2	W 62	2	217.3	0.1	43	8.9	0.37	0.91	48	42	248	
		7	41.9	0.1	9	7.6	1.01	0.83	53	61	232	
3	W 89	3	6.1	0.0	2	13.5	0.86	0.62	15	23	230	Day 7 229
		7	1.0	0.0	3	16.3	1.40	0.59	18	25	268	
4	M 56	3	115.7	0.1	38	8.5	1.54	0.87	48	100	361	
		9	5.5	0.1	4	8.6	2.20	0.81	40	125	234	
**Moderate CoD WHO 4**												
5	W 50	1	110.6	0.1	n.a	7.7	0.90	0.77	166	64	757	Day 1 573
		7	25.8	0.1	n.a	9.0	1.72	0.57	49	62	445	
6	M 53	1	113.8	1.5	40	3.8	0.51	1.26	165	81	797	
		6	89.3	0.3	114	5.6	0.92	1.21	114	143	585	
7	M 58	1	124.9	0.1	127	11.3	1.15	0.88	28	62	331	
		6	87.0	0.2	11	5.1	1.22	0.71	43	63	239	
8	M 50	2	124.6	0.4	94	24.1	12.59	1.08	124	65	549	Day 7 397
		9	6.4	0.1	4	14.8	10.72	0.83	71	144	378	
9	M 74	2	226.4	4.3	571	5.2	0.29	8.01	68	24	536	
		9	52.1	1.0	31	6.0	1.26	6.19	21	15	322	
10	W 69	2	111.6	1.4	48	10.0	1.51	1.91	164	42	470	
		10	1.9	0.0	67	13.4	3.20	0.52	45	54	415	
**Severe CoD WHO 5–7**												
11	W 88	3	86.5	0.5	191	3.9	0.51	2.58	28	13	227	Day 1 473
		9	35.9	1.9	47	3.9	0.60	1.43	33	10	324	
12	M 65	3	53.0	0.1	33	5.1	1.00	0.91	43	65	719	Day 7 622
		7	200.4	0.3	154	3.9	0.75	0.95	88	65	920	
**Reference values**	W M		< 5.0	< 0.1	< 8	4.4–11.3	1.26–3.35	0.50–0.90 0.50–1.10	< 35 < 50	< 35 < 50	< 250	

CoD = course of the disease. The age of the patients is reported in years.

### Recombinant SARS-CoV-2 proteins

We used recombinant SARS-CoV-2 S, R, S1, and O proteins to detect anti-SARS-CoV-2 antibodies in the serum of COVID-19 patients by immunoblot analysis. First, recombinant SARS-CoV-2 proteins were analyzed by SDS-PAGE following Coomassie staining and by immunoblotting (Fig. 1a,b). The S protein, which has a calculated molecular mass of 138.8 kDa, showed two bands with apparent molecular masses of 175 and 35 kDa in the Coomassie staining (Fig. 1a) and additional fragmentation bands in the immunoblot (Fig. 1b). To verify the presence of the fragmentation bands, we performed silver staining by SDS-PAGE (Fig. 1c). All immunoreactive bands (‘S up’ and ‘S low’) were considered when evaluating the immunoblots.

### Investigation of IgA, -M, and -G isotype antibodies by immunoblot analysis

To investigate the IgA, -M, and -G-specific antibody response of COVID-19 patients, we analyzed the patients’ serum by immunoblot. It should be noted that the Ig levels of the controls are used to define a patient as positive or negative. Furthermore, IgA, -M, and -G isotype antibodies with reactivity against SARS-CoV-2 proteins could also be detected in the controls (Supplementary Figs. S2–S4). Most patients did not show antibodies directed against all four SARS-CoV-2 proteins. The S and S1 proteins were the most commonly detected proteins, while the R protein was the least commonly detected (Table 2). Additionally, the antibody reactivity (signal intensity) was stronger against the S (S up) and S1 proteins than against the R and O proteins (Supplementary Figs. S2–S4). The presence and signal intensity of antibodies directed against the O protein varied between the different isotype classes (Table 2 and Supplementary Figs. S2–S4). However, IgM isotype antibodies directed against the O protein were detected in the majority of the patients. Only four out of 12 patients were positive for IgA isotype antibodies. In most patients, fewer IgA antibodies were detected than in the controls, so these patients were considered negative. Nevertheless, IgA antibodies with reactivity against SARS-CoV-2 proteins were present in the serum of all patients (Supplementary Fig. S3). Interestingly, IgA isotype antibodies in patients considered positive were reactive against the fragmented S protein (S low) but not the full-length S protein (S up). This is also true for IgM isotype antibodies. In contrast, IgG isotype antibodies were frequently directed against non-fragmented S protein (S up), while only four out of 12 patients showed these antibodies directed against the fragmented S protein (S low). All patient cohorts were positive for antibodies of almost all classes (Table 2). The only exception was IgA, which was negative in the patients in the severe group. To investigate the dynamics of the antibody response, we also analyzed the patients’ serum one week after admission to the hospital. As the disease progressed, patients showed more reactivity to anti-SARS-CoV-2 antibodies; reactivity decreased only in P5 for IgM, in P6 for IgA, and in P11 for IgG. However, P3 from the mild group was positive only for IgM antibodies on day 7, and P12 from the severe group was negative for all antibody classes at both time points. Unexpectedly, we found no correlation between the presence of antibodies and the future course of the disease. In all of the three groups –– mild, moderate, or severe –– there were patients with strong, weak, or no antibody reactivity.

**Table 2:** Evaluation of the patient immunoblots.

Patient	**Day 1**												**Day 7**											
	1	2	3	4	5	6	7	8	9	10	11	12	1	2	3	4	5	6	7	8	9	10	11	12
**IgM**																								
S up						+										+		+						
S low	+	+			+	+	+	+			+		+	+	+		+	+	+	+			+	
R	+					+							+											
S1	+				+		+						+			+			+			+		
O	+	+			+	+	+				+		+	+	+	+		+	+		+		+	
**IgA**																								
S up																								
S low										+												+		
R						+				+						+						+		
S1										+						+					+	+		
O										+								+				+		
**IgG**																								
S up	+	+		+		+	+	+	+	+	+		+	+		+	+	+	+	+	+	+	+	
S low	+					+					+		+				+	+	+					
R						+												+						
S1	+					+	+			+	+		+			+	+	+	+	+		+	+	
O	+					+					+		+				+	+	+					

The serum of COVID-19 patients 1–12, taken on the day of admission to the hospital (day 1) and again one week later (day 7), was used as a primary antibody to detect the recombinant SARS-CoV-2 proteins S, R, S1, and O by immunoblotting. The presence of anti-SARS-CoV-2 antibodies was determined for IgM, -A, and -G isotype antibodies. A patient was labeled positive for antibodies ( +) if the absolute band intensity of the patient exceeded the highest band intensity of the controls. All immunoblots are depicted in Supplementary Figs. S2–S4.

### ELISA

**Figure 2:**
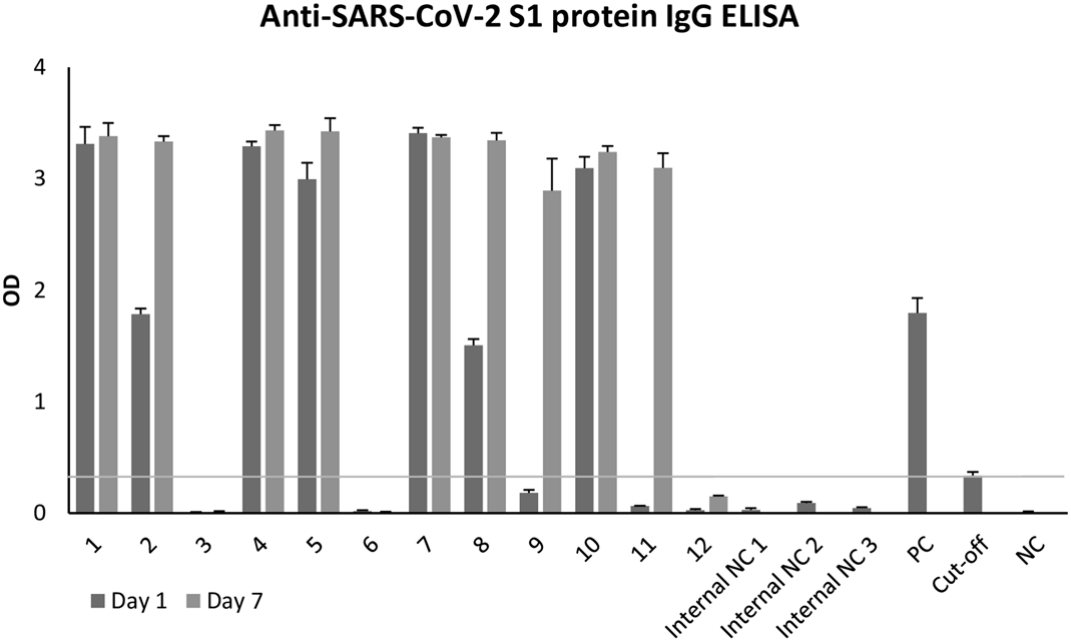
Anti-SARS-CoV-2 S1 protein IgG ELISA. Patient serum was tested for IgG isotype antibodies directed against subunit 1 of the SARS-CoV-2 spike protein (S1). Patients with a higher optical density (OD) measured at λ = 450 nm than the provided cut-off were labeled positive for IgG isotype antibodies directed against the S1 protein. Average ODs and standard deviations are shown in the bar chart. n = 3.

We analyzed the patients’ serum with a commercially available ELISA kit kindly provided by Immundiagnostik AG (Germany) (Fig. 2). The immunoassay detects human anti-SARS-CoV-2 S1 IgG isotype antibodies. The manufacturer provided positive and negative controls and a cut-off sample to facilitate the evaluation of the patients. By ELISA analysis, the serum of nine out of 12 patients was positive. Two of those were only positive on day 7 (P9 and P11), while the others were already positive on day 1. There was a general increase in signal intensity observable in patients on day 7. This increase was most prominent for P2, P8, P9, and P11. Conversely, P3 from the mild cohort, P6 from the moderate cohort, and P12 from the severe cohort showed no signals. The sera of the internal negative controls 1–3, which were used as cut-offs for the immunoblot analysis, were negative by ELISA.

### Comparison of immunoblot and ELISA analysis

Both immunoblotting and ELISA use the same recombinant S1 protein purified in the same manner. Strikingly, no anti-SARS-CoV-2 antibodies could be detected by either method in the serum of P12, and only a few IgM antibodies could be detected by immunoblot at day 7 in the serum of P3. However, the comparison of the immunoblot and ELISA results showed discrepancies. P6 and P11 on day 1 were positive for IgG isotype antibodies directed against the S1 protein in the immunoblot but not in the ELISA analysis. P2, P4, P5, P8, and P9 show IgG antibodies directed against the S1 protein on day 1 and day 7 by ELISA but not by immunoblotting. However, these patients showed IgG antibodies directed against the S protein in the immunoblot, indicating that anti-SARS-CoV-2 antibodies were present. ELISA (day 1: 7/12, day 7: 9/12; Table 3) showed more positive results for IgG isotype antibodies against the S1 protein than immunoblotting (day 1: 5/12, day 7: 8/12). By immunoblot analysis, most patients showed IgM antibodies directed against the S protein (day 1: 7/12, day 7: 9/12). Interestingly, patients also frequently showed IgM antibodies directed against the O protein (day 1: 6/12, day 7: 8/12). For IgG antibodies, only five out of 12 (day 1) and eight out of 12 (day 7) of the patients showed anti-S1 protein antibodies, while nine out of 12 (day 1) and ten out of 12 (day 7) showed anti-S protein antibodies (highest frequency). The R protein of SARS-CoV-2 was detected rather poorly in the immunoblot, indicating that there were only a few antibodies produced that are directed against this protein.

**Table 3:** Comparison of the results from the immunoblot and ELISA analysis.

	Immunoblot												ELISA
	IgM				IgA				IgG				IgG
	S	R	S1	O	S	R	S1	O	S	R	S1	O	S1
Positive Results Day 1 (n = 12)	7	2	3	6	1	2	1	1	9	1	5	3	7
Positive Results Day 7 (n = 12)	9	2	4	8	1	2	3	2	10	1	8	4	9

Positive results for each category were counted. n = 12.

## Discussion

The number of studies evaluating SARS-CoV-2 immunoassays is increasing steadily. However, immunoassays do not provide information about contamination or cross-reactivity, which might lead to false high sensitivity and lower specificity. Therefore, we investigated the serum of 12 COVID-19 patients by immunoblot analysis for the presence of IgM, -A, and -G isotype antibodies directed against the SARS-CoV-2 S, R, S1, and O proteins.

The most striking result of our study was the fact that we found no correlation between the presence of IgA, IgM, and IgG isotype antibodies and the future course of the disease. For example, a patient in the group with mild symptoms had no antibody reactivity against any isotype, whereas a patient in the severe group who finally died possessed IgA, IgM, and IgG isotype antibodies against SARS-CoV-2 proteins. Another striking result of the immunoblot analysis was the low number of patients who were positive for IgA isotype antibodies directed against SARS-CoV-2 proteins. This may be because serum is not the main localization of secreted, mature IgA isotype antibodies. However, a recent study showed that IgG, IgM, and to a lesser extent IgA responses to spike and RBD in serum positively correlated with matched saliva samples^12^. Nevertheless, our findings might also indicate an overall lack of IgA isotype antibodies in immune defense. This lack of IgA isotype antibodies in the respiratory tract could be a reason for SARS-CoV-2 reinfections^13,14^. On the other hand, the low number of patients positive for IgA isotype antibody in our study contradicts reports that describe an abundance of IgA isotype antibodies directed against the SARS-CoV-2 S protein in COVID-19 patients; reports which showed IgA had higher titers than IgM or even IgG isotype antibodies^15,16^. However, this contradiction can be explained by our set-up, as in this assay a patient was defined as positive or negative for IgA isotype antibodies according to the levels of IgA isotype antibodies detected in the controls. The presence of anti-SARS-CoV-2 IgA isotype antibodies in the controls is probably a result of cross-reactivity owing to previous infections with other coronaviruses or to random antibodies. In most patients, fewer IgA isotpe antibodies were detected than in the controls and consequently these patients were considered negative; nevertheless, IgA isotype antibodies with reactivity against SARS-CoV-2 proteins were present in the patients’ serum. To what extent these antibodies support immunity against the SARS-CoV-2 virus is unclear and can only be answered with functional, cell-based inhibition assays. This is true not only for IgA but also for IgM and IgG isotype antibodies. Based on the results of our immunoblot analysis, we assume that even patients who underwent COVID-19 or vaccination may have a negative immunoassay result when assessed against a threshold determined from controls who have immunity to the virus through existing antibodies.

A further, interesting result of our study is that IgM and -A isotype antibodies predominantly recognize the degradation product of the S protein (S low). Perhaps this degradation product is presented by the MHC II complex, which leads to higher antibody production against it. Interestingly, the S and S1 proteins were more frequently detected than the R protein. An explanation could be that the RBD of SARS-CoV-2 spike protein is usually not accessible, as it is masked by the rest of the protein^17^. Although antibodies against the RBD may be effective in inhibiting the virus from penetrating cells and may be suitable as a vaccine antigen^18^, the RBD alone is unsuitable as an antigen for diagnostic purposes. In contrast, the ORF8 protein is well detected through IgM antibodies but not by IgA or IgG antibodies. The function of ORF8 is still under investigation^19^. However, there are reports that it is involved in suppressing the host’s immune response through the downregulation of MHC I complexes^20^ and suppressing interferon type I^21^. Thus, ORF8 is a promising pharmaceutical target but is not suitable for detecting IgA and -G antibodies.

We found differences between the results of the immunoblot and ELISA analysis of IgG isotype antibodies directed against the S1 protein. Sometimes, antibodies were detected by immunoblot but not by ELISA, and vice versa. The virus proteins are denatured for the immunoblot, while the ELISA utilizes their native form; therefore, the ELISA detects three-dimensional epitopes whereas the immunoblot detects linear ones, which might lead to differing results. According to the immunoblot analysis, the S1 protein is not necessarily the best antigen for detecting IgM isotype antibodies, as more patients were positive for antibodies directed against the S protein. Using a combination of the S protein and the O protein could prove to be best for detecting IgM isotype antibodies, as the O protein is a potent antigen for detecting COVID-19 patients in the early to late stages of infection^22^. When detecting IgG isotype antibodies, our data suggest that the use of the S protein should be sufficient to identify positive patients. However, there is evidence that the use of the S1 domain instead of the full-length S protein leads to less cross-reactivity^23^.

Based on our results, we wondered if the presence of antibodies could help to stratify the patients, but we could not observe any correlation between the presence of anti-SARS-CoV-2 antibodies and the course of the disease. We found high levels of the inflammatory parameters CRP and IL-6, but not of leukocyte count or PCT, and found a rise in LDH level with the severity of the disease. In the context of COVID-19 disease, an excessive inflammatory response, also called hyperinflammatory syndrome, can occur. Patients with hyperinflammatory syndrome may benefit from therapy targeting cytokine storm syndrome. Recently, a set of clinical criteria for COVID-19-associated hyperinflammatory syndrome was proposed^24^. Furthermore, we observed a correlation between CRP and IL-6 as well as lymphocyte count and antibody reactivity. Within one week, CRP and IL-6 levels decreased, while lymphocyte counts and antibody reactivity increased, in patients 1–11. Conversely, for P12, who was not immunosuppressed and showed no antibody reactivity, the lymphocyte count decreased, while CRP and IL-6 levels increased. However, we cannot provide any explanation for why the patients in the severe cohort showed lower values for CRP and IL-6 than those in the moderate and mild cohorts. Perhaps the lower values are a result of a compensatory anti-inflammatory response.

A limitation of our study is the small number of patients and controls. The inclusion of more patients and a better match in the age of the patients (median age of 59 years) and of the control donors (median age of 38 years) would have increased the statistical power of this study. In our study, we found no correlation between the presence of antibodies and the future course of the disease. We cannot exclude the possibility that a correlation could be seen if we included more patients. Nevertheless, we could show that only the presence or absence of antibodies is not decisive for the course of the disease. Further, a study with more patients would most certainly have confirmed the trend of IgM and IgG isotype antibodies to show reactivity more often against the full-length spike (S) protein than against the subunit 1 (S1) or the receptor-binding domain (R) (Table 3). Furthermore, even in our small patient population, the O protein is less immunogenic than the S protein (Table 3), and it is very likely that this trend would be confirmed with an increased number of patients. The influence of the inclusion of more controls is shown in the case of IgA isotype antibodies. Here, one control (NC3; supplementary Fig. S3) showed a pronounced antibody reaction directed against the S and O protein. Thus, the threshold used to define a patient as positive for IgA isotype antibodies was much higher than for IgM and IgG isotype antibodies. Accordingly, fewer patients were defined as positive for IgA than for IgM and IgG antibodies. This leads also to a discrepancy between our study and other studies which report a higher number of patients positive for IgA isotype antibodies^15,16^. With the inclusion of more controls, some controls would show pronounced reactivities of IgG and IgM isotype antibodies against SARS-CoV-2 proteins, which would raise the threshold and lead to a lower number of patients defined as positive for IgM and IgG isotype antibodies. Regardless of the statistical strength of our study, we were able to show the presence of preformed and/or cross-reacting IgM, IgA, and IgG isotype antibodies against SARS-CoV-2 proteins. Further, a negative test result for antibodies directed against the SARS-CoV-2 proteins does not equate to the complete absence of antibodies. A person with a negative immunoassay test result may still have SARS-CoV-2 antibodies and therefore possesses immunity or at least partial immunity against the virus.

In conclusion, we found evidence of preformed antibodies or antibody cross-reactivity, which questions the reliability of negative results from immunoassays detecting anti-SARS-CoV-2 antibodies in serum samples. Nevertheless, for the detection of anti-SARS-CoV-2 IgG antibodies, our data suggest that the use of the S protein in immunoassays should be sufficient to identify positive patients. Using a combination of the S protein and the O protein could prove to be the best method of detecting patients that are positive for anti-SARS-CoV-2 IgM antibodies. Unexpectedly, we found no correlation between the presence of antibodies and the future course of the disease. To assess the severity of a SARS-CoV-2 infection, attention should be paid to the parameters CRP, IL-6, and LDH.

## Supplementary Information

Supplementary Information

## Data Availability

All data generated or analyzed during this study are included in this published article (and its Supplementary Information files).
