# Supplementary material for: Analysis of IgM, IgA, and IgG isotype antibodies Directed against SARS-CoV-2 spike glycoprotein and ORF8 in the course of COVID-19
Source: Sci Rep. 2021 Apr 26;11:8920. doi: 10.1038/s41598-021-88356-8 (PMC8076237; doi:10.1038/s41598-021-88356-8)
Supplement: Supplementary file 1 — Supplementary Information [file 41598_2021_88356_MOESM1_ESM.docx]

**Analysis of IgM, IgA, and IgG Isotype Antibodies Directed Against SARS‑CoV‑2 Spike Glycoprotein and ORF8 in the Course of COVID‑19.**

Denise Meinberger^1^, Manuel Koch^2,3,4^, Annika Roth^1^, Gabriele Hermes^1^, Jannik Stemler^5, 6, 7^, Oliver A. Cornely^5,6,7^, Thomas Streichert^1^, Andreas R. Klatt^1^_._

Supplementary Information

Supplementary Table S1: Primer and restriction enzymes. Restriction sites are marked with capital letters.

| Protein | Primer sequence | Restrictionenzyme |
| --- | --- | --- |
| Spike protein (S) fw | ccgaCGTCTCaagcttctgggcaacgtgctggttattg | Esp3I |
| Spike protein (S) rev | cggtGGATCCttgttcatacttccccaactc | BamHI |
| RBD (R) fw | acaGCTAGCgtttatgcgtggaatcgcaaac | NheI |
| RBD (R) rev | ggtGGATCCttagaccgtggccggtgcgtgcaaaag | BamHI |
| Subunit 1 (S1) fw | accaTCTAGAacagtgcgtgaatcttaccac | XbaI |
| Subunit 1 (S1) rev | cagtGGTCTCagatccttattttttcggaccacagaccgtg | BsaI |
| ORF8 (O) fw | accaGCTAGCgcatttcaccaagaatgtag | NheI |
| ORF8 (O) rev | ggtGGATCCttagatgaaatctaaaacaac | BamHI |


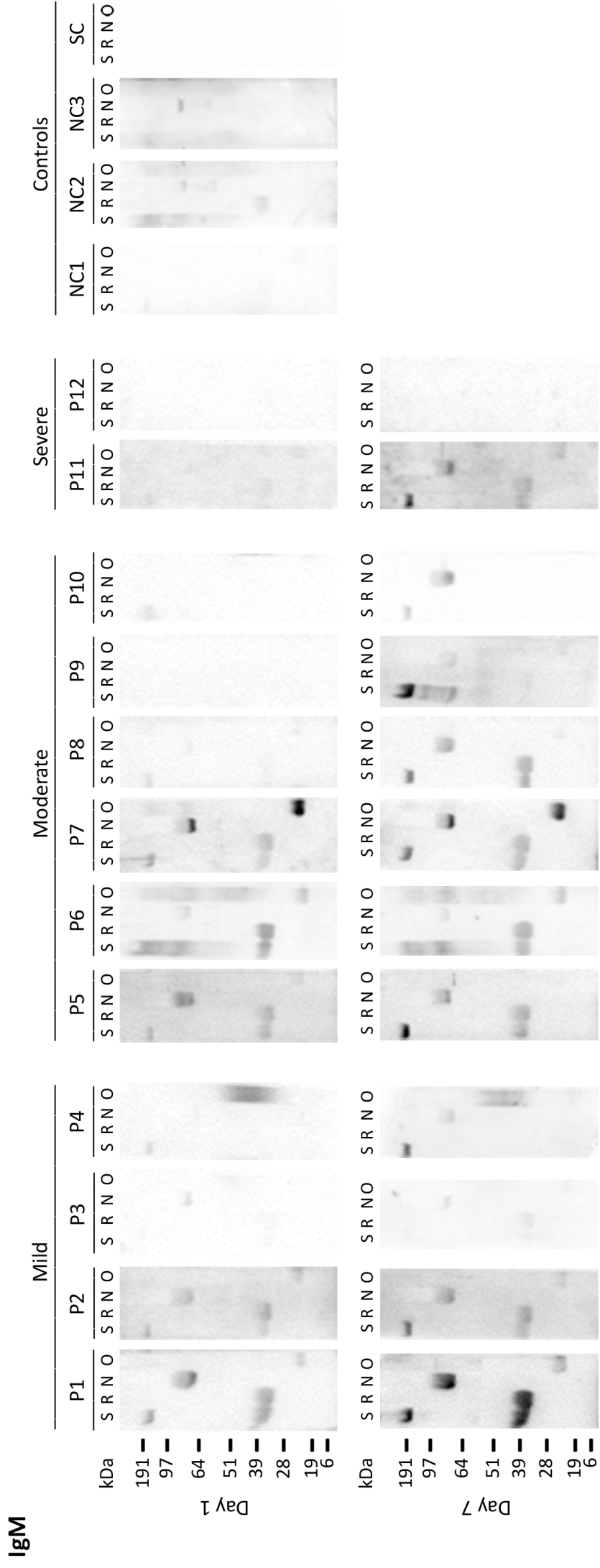


Supplementary Figure S2: Immunoblot analysis of anti‑SARS‑CoV‑2 IgM isotype antibodies.

Immunoblot with 2 µg of recombinant SARS‑CoV‑2 spike protein (S), the RBD of spike protein (R), the subunit 1 of the spike protein (S1), and ORF8 protein (O) incubated with patient´s serum and detected with an anti‑human IgM HRP‑conjugated antibody. Cropped lanes of different immunoblots are divided by white space. Immunoblots were only cut in width and lanes are shown in their full-length. All immunoblots are displayed with the same exposure time.


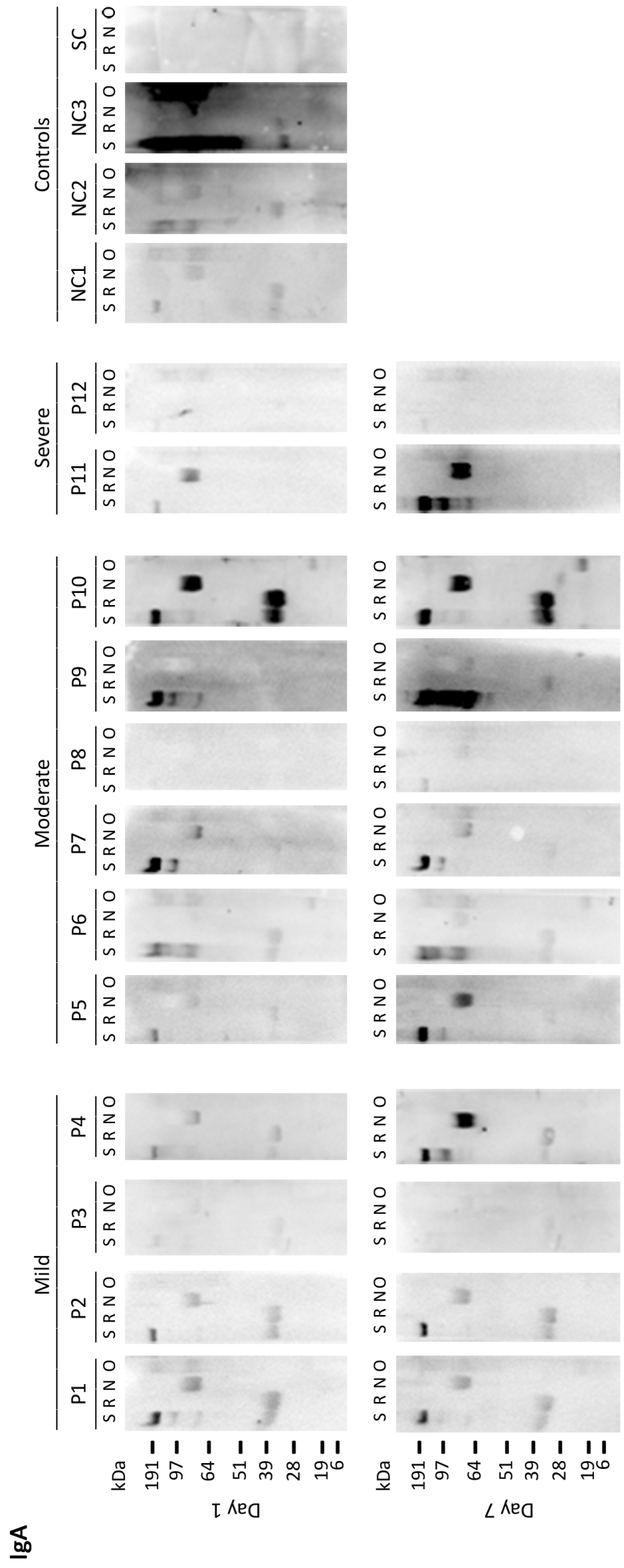


Supplementary Figure S3: Immunoblot analysis of anti‑SARS‑CoV‑2 IgA isotype antibodies.

Immunoblot with 2 µg of recombinant SARS‑CoV‑2 spike protein (S), the RBD of spike protein (R), the subunit 1 of the spike protein (S1), and ORF8 protein (O) incubated with patient´s serum and detected with an anti‑human IgA HRP‑conjugated antibody. Cropped lanes of different immunoblots are divided by white space. Immunoblots were only cut in width and lanes are shown in their full-length. All immunoblots are displayed with the same exposure time.


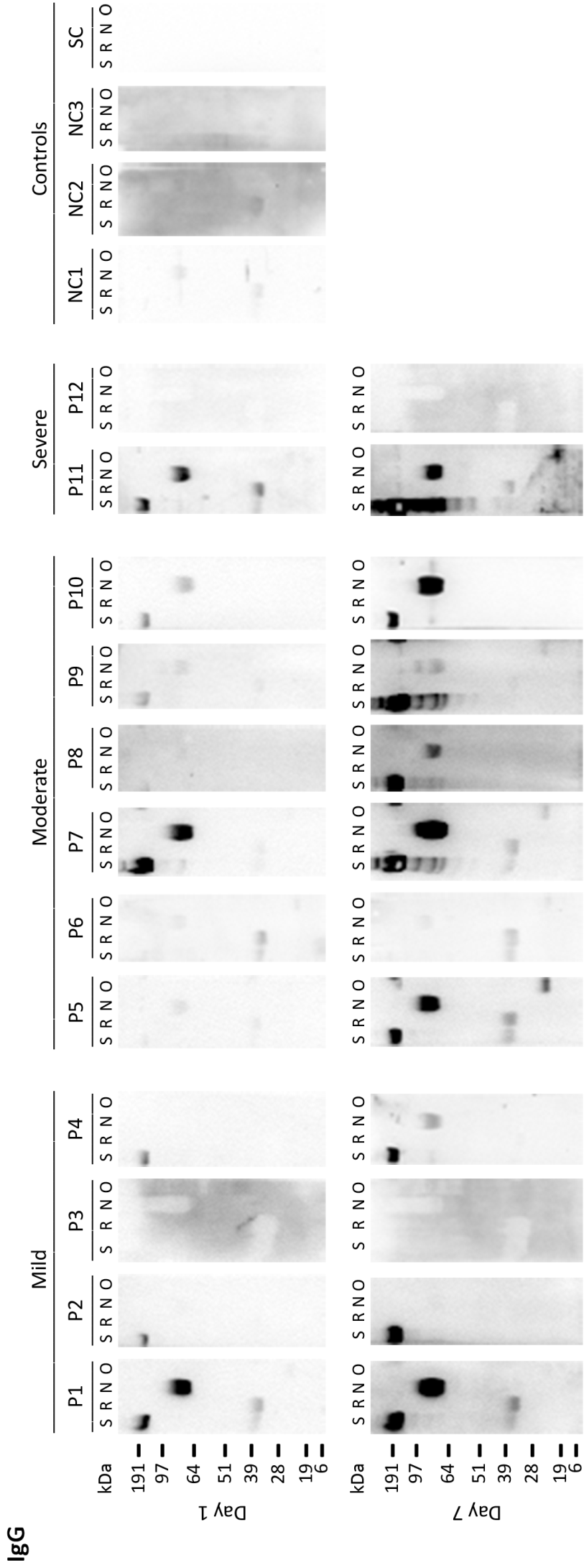


Supplementary Figure S4: Immunoblot analysis of anti‑SARS‑CoV‑2 IgG isotype antibodies.

Immunoblot with 2 µg of recombinant SARS‑CoV‑2 spike protein (S), the RBD of spike protein (R), the subunit 1 of the spike protein (S1), and ORF8 protein (O) incubated with patient´s serum and detected with an anti‑human IgG HRP‑conjugated antibody. Cropped lanes of different immunoblots are divided by white space. Immunoblots were only cut in width and lanes are shown in their full-length. All immunoblots are displayed with the same exposure time.
